# A protocol to differentiate the chondrogenic ATDC5 cell‐line for the collection of chondrocyte‐derived extracellular vesicles

**DOI:** 10.1002/jex2.70004

**Published:** 2024-09-05

**Authors:** Jose G. Marchan‐Alvarez, Loes Teeuwen, Doste R. Mamand, Susanne Gabrielsson, Klas Blomgren, Oscar P. B. Wiklander, Phillip T. Newton

**Affiliations:** ^1^ Department of Women's and Children's Health Karolinska Institutet Stockholm Sweden; ^2^ Astrid Lindgren Children's hospital Stockholm Sweden; ^3^ Division of Immunology and Allergy, Department of Medicine (Solna) Karolinska Institutet Stockholm Sweden; ^4^ Clinical Immunology and Transfusion Medicine Karolinska University Hospital Stockholm Sweden; ^5^ Department of Laboratory Medicine Unit for Biomolecular and Cellular Medicine Karolinska Institutet Stockholm Sweden; ^6^ Pediatric Oncology Karolinska University Hospital Stockholm Sweden

**Keywords:** ATDC5 cells, bone fracture healing, endochondral ossification, extracellular vesicles, Opti‐MEM

## Abstract

Skeletal growth and fracture healing rely on the mineralization of cartilage in a process called endochondral ossification. Chondrocytes firstly synthesize and then modify cartilage by the release of a wide range of particles into their extracellular space. Extracellular vesicles (EVs) are one type of such particles, but their roles in endochondral ossification are yet to be fully understood. It remains a challenge to obtain representative populations of chondrocyte‐derived EVs, owing to difficulties both in preserving the function of primary chondrocytes in culture and in applying the serum‐free conditions required for EV production. Here, we used the ATDC5 cell‐line to recover chondrocyte‐derived EVs from early‐ and late‐differentiation stages, representing chondrocytes before and during cartilage mineralization. After screening different culture conditions, our data indicate that a serum‐free Opti‐MEM‐based culture medium preserves chondrocyte identity and function, matrix mineralization and cell viability. We subsequently scaled‐up production and isolated EVs from conditioned medium by size‐exclusion chromatography. The obtained chondrocyte‐derived EVs had typical ultrastructure and expression of classical EV markers, at quantities suitable for downstream experiments. Importantly, chondrocyte‐derived EVs from late‐differentiation stages had elevated levels of alkaline phosphatase activity. Hence, we established a method to obtain functional chondrocyte‐derived EVs before and during cartilage mineralization that may aid the further understanding of their roles in endochondral bone growth and fracture healing.

## 1 INTRODUCTION

During childhood and adolescence, we increase in height due to the longitudinal growth of the tubular bones, particularly those in the legs and spine. These bones grow via a cartilage precursor in a process known as endochondral ossification: within the hyaline cartilage that they secrete, chondroprogenitor cells generate proliferating chondrocytes that differentiate into hypertrophic chondrocytes, which re‐model and calcify their extracellular matrix (ECM). Eventually, the majority of hypertrophic chondrocytes undergo cell death, and the mineralized cartilage is used as a scaffold on which osteoblasts deposit new bone tissue in the primary spongiosa (Chagin & Newton, 2020). This process can be recapitulated during the healing of bone fractures whereby a cartilage callus precedes bone formation (Einhorn & Gerstenfeld, 2015). Chondrocyte activity, differentiation and mineralization are tightly regulated throughout endochondral ossification by diverse stimuli, including particles secreted by the chondrocytes (Arnold et al., 2021; Hallett et al., 2019; Kozhemyakina et al., 2015).

Extracellular vesicles (EVs) are double‐membraned, fluid‐filled sacs that are released by all cells (Wiklander et al., 2019). EVs are involved in many processes including intercellular communication (Couch et al., 2021), disposal of waste material (Couch et al., 2021) and ECM mineralization (Anderson, 1969; Bonucci, 1967). Cells release EVs containing a variety of biologically active cargo molecules, such as lipids, proteins, and nucleic acids, and thereby have the potential to regulate core biological processes ranging from tissue homeostasis to pathophysiological events (Wiklander et al., 2019). These nano‐sized particles can be classified into three main subsets based on their biogenesis, size and composition: exosomes (50–150 nm diameter) that arise during the fusion of multivesicular bodies with the plasma membrane (van Niel et al., 2022), ectosomes (100–1000 nm diameter) that form by outward budding from the plasma membrane (van Niel et al., 2022) and the less studied sub‐group known as apoptotic bodies (100–5000 nm diameter) that emerge from the orderly fragmentation of apoptotic cells (Li et al., 2020).

Originally described as matrix vesicles, chondrocyte‐derived EVs from the growth plate have been regarded as initiators of calcium phosphate crystals in the luminal side of the vesicles, leading to mineralization and subsequently long‐bone elongation (Anderson, 1969; Bonucci, 1967). Importantly, recent reports have interrogated the role of these chondrocyte‐derived EVs in cellular signalling and cell‐cell communication *in vitro* (Asmussen et al., 2021; Boyan et al., 2022; Lin et al., 2018). Interestingly, depending on the cell maturation state, matrix vesicles have different molecular compositions (e.g., lipids, enzymes, growth factors, hormones and microRNAs), suggesting they may regulate endochondral bone formation via transfer of specific cargos, such as microRNAs (Lin et al., 2018).

A major challenge to chondrocyte researchers is that primary chondrocytes are prone to de‐differentiation when cultured *ex vivo* (Chen et al., 2022); therefore, large‐scale production of EVs from primary cells is not feasible. Alternatively, approaches to obtain EVs using chondrocyte cell lines, with a more stable phenotype and function, are yet to be established. Thus, EV production and isolation from stable chondrogenic sources is a key challenge in the field. The murine ATDC5 cell line is one of the most well‐characterized tools used to obtain and study chondrocytes *in vitro* (Newton et al., 2012; Yao & Wang, 2013), and is, therefore, a suitable model from which to obtain chondrocyte‐derived EVs for downstream applications.

Like many cell lines, ATDC5 cell differentiation protocols rely on fetal bovine serum (FBS) (Shukunami et al., 1997). However, since EVs isolated from cultured cells are extracted from conditioned media, the exogenous EVs present in FBS (originating from the donor animals) would represent a contaminant to the chondrocyte‐derived EVs. Therefore, for EV isolation, it is typical that cells are cultured in two different media compositions, sequentially: serum‐containing‐media followed by serum‐free media (Bost et al., 2022). Whereas the former provides crucial components to promote normal cell growth and differentiation, the latter allows the collection of EVs deriving from the cells‐of‐interest. However, serum removal may alter cell properties, which can in turn impact EV quantity, cargo and function (Li et al., 2015). Thus, it is important to verify that EV collection media do not cause abnormal cell behaviors in order to obtain EV populations that represent those deriving from the parental cells.

**FIGURE 1:**
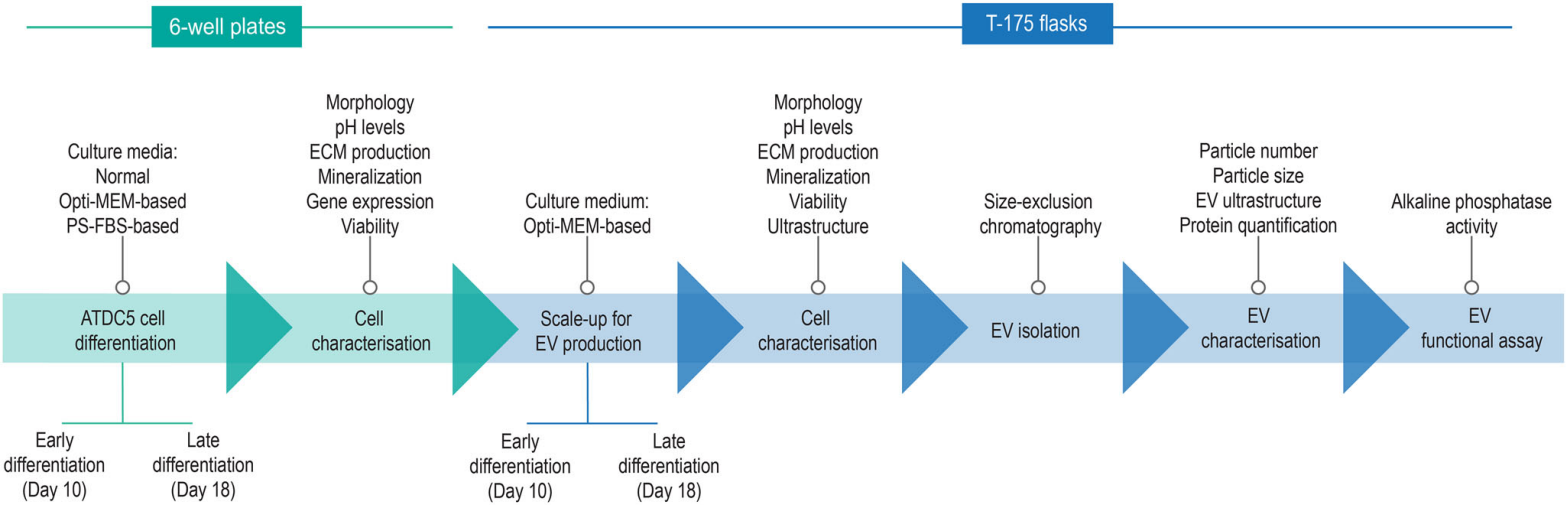
Schematic illustration of the experimental workflow used to produce EVs from chondrocyte cultures at early‐ and late‐differentiation stages. Initial experiments were performed using several types of culture media to assess cell differentiation in six‐well plates (green); subsequently, the selected medium was used to scale‐up the process in T‐175 flasks and characterization was extended to the obtained EVs (blue). ECM, extracellular matrix.

Here, we set out to establish a protocol to isolate chondrocyte‐derived EVs from ATDC5 cells. To identify a suitable EV collection medium, we first compared custom EV‐depleted media with normal growth conditions at an early (day 10, prior to ECM mineralization) and a late (day 18, during ECM mineralization) differentiation stage (Figure 1). All conditions were compared in terms of phenotype, variations in conditioned media pH, histochemical staining, gene expression and cell viability. We determined that a chondrogenic Opti‐MEM‐based culture medium was the most suitable medium based on its ability to induce a typical chondrogenic differentiation, whilst maintaining cell viability. Next, we scaled‐up the devised method and confirmed the functionality and viability of these chondrocytes at a cellular and ultrastructural level (Figure 1). Thereafter, EVs were isolated using size‐exclusion chromatography (SEC) and EV characteristics, including particle size, ultrastructure and protein:particle ratio, were assessed by a variety of techniques. A functional comparison between early‐ and late‐chondrocyte‐derived EVs revealed that late‐chondrocyte‐derived EVs harboured elevated mineralization capacity. Notably, this approach takes advantage of the benefits of SEC, which is a well‐established method to isolate EVs and maintain integrity and functionality (O'Brien et al., 2022). Together, these data demonstrate the ability of the protocol to generate chondrocyte‐derived EVs with structural and functional characteristics of their *in vivo* counterparts. Hence, our research provides a novel approach to obtain high‐quality chondrocyte‐derived EVs for studies focused on bone research. Of note, this research was performed according to the general principles of EV experimentation outlined in the MISEV2018 guidelines (Théry et al., 2018).

## 2 MATERIALS AND METHODS

To contribute to the standardization of the EV field, we have submitted all relevant data of our experiments to the EV‐TRACK knowledgebase (EV‐TRACK ID: EV231012) (Van Deun et al., 2017).

### 2.1 Standard ATDC5 culture conditions

Chondrogenic ATDC5 cells (Riken Cell Bank, Ibaraki, Japan) were cultured as previously reported (Newton et al., 2012). Briefly, cells were grown in DMEM/F‐12 (Dulbecco's Modified Eagle Medium/ Hams F‐12) containing GlutaMAX (Gibco, catalogue number: 31331093) and supplemented with 5% FBS (Gibco, catalogue number: 10082147), 1% insulin‐transferrin‐selenium (Gibco, catalogue number: 41400045), 1% sodium pyruvate (Gibco, catalogue number: 11360070) and 0.5% gentamicin (Gibco, catalogue number: 15750045), at a density of 6000 cells/cm^2^ in six‐well plates (TPP, catalogue number: Z707759). Cells were left for 6 days to reach confluency. From this point onwards, the medium was supplemented with 10 mM β‐glycerophosphate (BGP) (Sigma–Aldrich, catalogue number: G9422) and 50 µg/mL L‐ascorbate‐2‐phosphate (ascorbic acid) (Sigma–Aldrich, catalogue number: G5960), herein known as normal culture medium (Table 1). Cell cultures were maintained in a humidified atmosphere (37°C, 5% CO_2_) and the medium was changed every 2–3 days. All cell cultures were performed using a passage between 17 and 20.

**TABLE 1:** Media composition for ATDC5 cell cultures.

Normal	Opti‐MEM‐based	PS‐FBS‐based
DMEM/F‐12 (92.5%)	Opti‐MEM (97.5%)	DMEM/F‐12 (92.5%)
FBS (5%)	–	PS‐FBS (5%)^a^
ITS (1%)	ITS (1%)	ITS (1%)
Sodium pyruvate (1 mM)	Sodium pyruvate (1 mM)	Sodium pyruvate (1 mM)
Gentamycin (0.5%)	Gentamycin (0.5%)	Gentamycin (0.5%)
BGP (10 mM)	BGP (10 mM)	BGP (10 mM)
AA (50 ug/mL)	AA (50 ug/mL)	AA (50 ug/mL)

Abbreviations: AA, L‐ascorbate‐2‐phosphate (ascorbic acid); BGP, beta‐glycerophosphate; FBS, fetal bovine serum; ITS, insulin transferrin selenium.

^a^ PS‐FBS was prepared by ultracentrifugation (please, see experimental section).

### 2.2 Preparation of pre‐spun FBS (PS‐FBS)

Since FBS contains its own population of EVs, it was necessary to dilute and centrifuge the FBS to produce a pre‐spun FBS form (Théry et al., 2006). For this, the FBS was diluted (1:3) in DMEM/F‐12 containing GlutaMAX, aliquoted in ultra‐clear tubes (Beckman Coulter, catalogue number: 344059) and subjected to ultracentrifugation (100,000×*g* for 18 h at 4°C) using the Optima L‐90K ultracentrifuge (rotor SW40). Subsequently, the pre‐spun FBS was filtrated (0.22 µm), aliquoted and stored at −20°C until further use.

### 2.3 Evaluation of the effect of different serum‐free media on chondrogenic differentiation

Cells were cultured for 8 or 16 days as per 2.1. Thereafter, cells were washed with DMEM/F‐12 containing GlutaMAX and then exposed to a chondrogenic Opti‐MEM‐based medium (Table 1) or chondrogenic PS‐FBS‐based medium (Table 1) for 48 h. The chondrogenic Opti‐MEM‐based medium comprised Opti‐MEM culture medium (Gibco, catalogue number: 31985047) supplemented with 1% insulin‐transferrin‐selenium, 1% sodium pyruvate and 0.5% gentamicin, BGP (10 mM) and ascorbic acid (50 µg/mL), and without FBS. The chondrogenic PS‐FBS culture medium was prepared using DMEM/F‐12 containing GlutaMAX and supplemented with 1% insulin‐transferrin‐selenium, 1% sodium pyruvate and 0.5% gentamicin, BGP (10 mM), and ascorbic acid (50 µg/mL) and 5% PS‐FBS, as described in Section 2.2. For clarity, side‐by‐side comparisons are presented in Table 1.

### 2.4 Scaling‐up the EV production from chondrocyte cultures

ATDC5 cells were cultured in T‐175 flasks (Sarstedt, catalogue number: 83.3912.002) as described in Section 2.1. Chondrocyte differentiation was maintained up to day 8 or day 16. At these time‐points, cells were washed with DMEM/F‐12 containing GlutaMAX and grew in 36.5 mL of chondrogenic Opti‐MEM‐based culture medium (Table 1) for 48 h. Subsequently, the 48‐h conditioned media was collected for EV isolation, and cell characterization was performed. In total, we generated six batches of EVs from each time‐point (early‐ and late‐differentiation), where each batch consisted of five T‐175 flasks. Each batch represents an independent differentiation experiment.

### 2.5 Mycoplasma test

Cells were routinely screened for mycoplasma according to the manufacturer´s instructions (Bionordika, Lonza, catalogue number: LZ‐LT07‐518); results were always negative.

### 2.6 Cell viability

Cell viability was assessed using calcein AM (Invitrogen, catalogue number: C3099) and propidium iodide (Thermo Scientific Chemicals, catalogue number: J66764.MC). Briefly, calcein AM (2 µM) and propidium iodide (2 µg/mL) were added directly to the wells and incubated on cells for 15–30 min. Thereafter, samples were observed under a fluorescent microscope (Invitrogen AMEFC4300). The live cell quantification was subsequently performed by Hoechst (Molecular Probes/Life Technologies, catalogue number: H3570) and propidium iodide. For this, Hoechst (10 mg/mL) and propidium iodide (2 µg/mL) were applied to the cell cultures and incubated for 15–30 min and finally samples were visualized under a fluorescent microscope. Quantification of the live cells was carried out using ImageJ analysis software (Schneider et al., 2012).

### 2.7 Fluctuations in extracellular pH

Freshly‐prepared media aliquots and conditioned media from cell cultures were analysed using a pH meter (VWR, pHenomenal).

### 2.8 Histological staining for proteoglycan deposition using Alcian blue staining

Cells were washed three times with PBS (1X) and then fixed in 95% methanol (Fisher Scientific, catalogue number: 10532971) for 20 min and stained with 1% Alcian blue 8GX (Sigma–Aldrich, catalogue number: A5268) in 0.1 M HCl overnight, as described previously (Newton et al., 2012). Thereafter, cells were washed in distilled water (dH_2_0) and images were captured. 6 M guanidine‐HCl (Thermo Scientific, catalogue number: 24115) was applied to the cell cultures for 6 h at room temperature to extract the Alcian blue and the optical density (OD) was determined at 630 nm by spectrophotometry (BMG Labtech, FLUOstar omega). Blanks (6 M guanidine‐HCl) were included as negative controls.

### 2.9 Evaluation of mineralization of ATDC5 cells by alizarin red staining

Cell cultures were washed three times with PBS (1X), fixed in 4% paraformaldehyde and stained with 2% alizarin red (Sigma–Aldrich, catalogue number: A5533), diluted in dH_2_O and pH = 4.2 (calibrated using HCl), for 5 min at room temperature. Cells were washed with dH_2_O and images collected. Alizarin red‐stained cultures were extracted with 10% cetylpyridinium chloride (Sigma–Aldrich, catalogue number: C0732) for 10 min and the OD was measured at 570 nm by spectrophotometry as per 2.8. Blanks (10% cetylpyridinium chloride) were included as negative controls.

### 2.10 Quantitative real‐time, reverse‐transcription PCR (qRT‐PCR)

RNA was extracted using the RNeasy Mini Kit (Qiagen, catalogue number: 74104) according to the manufacturer's instructions. RNA was reverse transcribed using Supertranscript III RT (200 U/µL) (Invitrogen, catalogue number: 18080044). Quantitative PCR was performed with the TaqMan Fast Universal Master Mix (Applied Biosystems, catalogue number: 4444556) and monitored with the Biorad‐96 Real‐Time PCR System. TaqMan primers were obtained from Thermo Fisher Scientific. Expression of *Sox9* (mouse, Mm00448840_m1), *Col2a1* (mouse, Mm01309565_m1), *Col1a1* (mouse, Mm00801666_g1) and *Col10a1* (mouse, Mm00487041_m1) were analysed, whereas *Gapdh* (mouse, Mm99999915_g1) was selected as a housekeeping gene. Data were normalized to *Gapdh* and to the control condition (ΔΔCt = ([Ct_gene of interest_‐Ct_GAPDH_) sample‐ (Ct_gene of interest_‐Ct_GAPDH_] control). Please note that qRT‐PCR experiments for each time‐point were run separately, precluding direct comparison. Values were reported as relative gene expression normalized to the control (Normal medium group on Day 10 for each gene).

### 2.11 EV isolation by SEC

Initially, cell culture‐derived conditioned media was pre‐cleared of cells and larger debris using two consecutive centrifugations (700×*g* for 5 min at 4°C followed by 2000×*g* for 10 min at 4°C in a Rotina 420R centrifuge). Thereafter, the supernatant was filtered through 0.22 µm bottle top vacuum filters (Nalgene Rapid‐Flow, catalogue number: 568‐0020) to remove any remaining larger particles. The filtered supernatant was then concentrated (3000×*g* for 30 min at 4°C) using Amicon Ultra 100K centrifugal filters (Millipore, catalogue number: UFC9100). The samples were subsequently loaded on qEV Gen 2 columns (70 nm, Izon Science) and the EV‐enriched fractions (fractions 7 to 10) were collected and pooled (∼2 mL) according to the manufacturer's instructions. Vivaspin 2 (MWCO 10 kDa) spin‐filters (Merck, catalogue number: Z614238) were used to concentrate the sample to a final volume of ∼100 µL. EVs were finally stored using Maxymum Recovery polypropylene 1.5 mL tubes (Corning, Axygen Maxymum Recovery, catalogue number: MCT‐150‐L‐C) and in 0.22 µm filtered phosphate‐buffered saline (PBS) supplemented with HEPES (ThermoFisher, catalogue number: 15630‐080), bovine serum albumin (Sigma–Aldrich, catalogue number: 05470), and trehalose [Sigma–Aldrich, D‐(+)‐Trehalose dihydrate, catalogue number: T0167‐25G], hereafter known as PBS‐HAT (1X), as described recently (Görgens et al., 2022). Please note that the human albumin used in the original protocol was replaced with bovine serum albumin.

### 2.12 Nanoparticle tracking analysis (NTA)

To determine the particle concentration and size from each batch of EVs, and PBS‐HAT (1X), NTA was applied. All samples were diluted in 0.22 µm filtered PBS (1X) and characterized with a Nanosight LM10‐HSGF instrument equipped with a 488 nm laser (Malvern Panalytical Ltd) and NTA 3.0 analytical software (Malvern Panalytical Ltd). Five 30‐s videos were recorded for each sample in light scatter mode with standard camera settings (level 13) at room temperature, while injecting the sample with a syringe pump (speed 50). Software settings were kept constant for all measurements (screen gain 10, detection threshold 3).

### 2.13 Protein quantification in EV preparations

Protein concentration was assessed using the micro‐BCA protein assay kit (Thermo Scientific, catalogue number: 23235). Please note that the chondrocyte‐derived EVs did not contain any PBS‐HAT for the purpose of this analysis. Briefly, 5 uL of the chondrocyte‐derived EVs were added to 145 uL of the Micro BCA working reagent. The contents of one albumin standard ampule (provided by the manufacturer) were diluted to determine the protein concentration of each unknown sample using a standard curve. Absorbance was determined at 562 nm by spectrophotometry as per 2.8. By dividing the particle number, obtained from the NTA, by the protein concentration from freshly‐isolated EVs, obtained from the micro‐BCA assay, we calculated the protein:particle ratio.

### 2.14 Transmission electron microscopy (TEM) of chondrocytes cultured in T‐175 flasks

Thermanox plastic coverslips for cell culture (Thermo Scientific Nunc Thermanox, catalogue number: 174950) were attached to the plastic surface of T‐175 flasks using 50 µL of rat tail collagen (Thermo Scientific, catalogue number: A1048301) and incubated for 24 h (37°C, 5% CO_2_) to allow the coverslip to adhere to the plastic surface. At the end of the 24‐h incubation period, ATDC5 cells were seeded into these T‐175 flasks and then cultured to both differentiation time‐points (day 10 and day 18), as described in Section 2.4. Eventually, chondrocytes covered the plastic surface including the Thermanox coverslips. Next, cell cultures at day 10 or day 18 were washed three times with PBS (1X). Thereafter, the Thermanox coverslips were recovered and immersed in fixation solution (2.5% glutaraldehyde [Ladd Research Industries, catalogue number: 20105] in 0.1 M phosphate pH 7.4) for 1 h at room temperature, and stored at 4°C. The coverslips were subsequently rinsed in 0.1 M phosphate buffer and post‐fixed in 2% osmium tetroxide (TAAB, catalogue number: O018) in 0.1 M phosphate buffer, pH 7.4 at 4°C for 2 h. Following stepwise dehydration in ethanol and acetone, the coverslips were embedded in LX‐112 resin (Ladd). Ultrathin sections (approximately 80–100 nm) were prepared using an EM UC7 (Leica), incubated with uranyl acetate (TAAB, catalogue number: U001) followed by lead citrate (Merck, catalogue number: 7398) and finally examined in an HT7700 transmission electron microscope (Hitachi High‐Technologies,) at 80 kV. Digital images were acquired using a 2kx2k Veleta CCD camera (Olympus Soft Imaging Solutions).

### 2.15 TEM of EVs

EVs isolated by SEC were subjected to negative staining for TEM. In brief, an aliquot of 5 µL from each sample was added to a grid with a glow discharged carbon coated supporting film for 1 min. The excess solution was removed with a filter paper, and the grid was incubated with 5 µL 1% uranyl acetate in water for 7 s. Excess stain was removed with a filter paper and the grid air‐dried. The samples were then examined in Talos 120 C G2 (Thermo Scientific) equipped with a CETA‐D detector at 120 kV.

### 2.16 Assessing the EV markers by Western blot

A total of 2 × 10^10^ EVs, and 2 × 10^6^ chondrocytes were lysed using 100 µL of radioimmunoprecipitation buffer (RIPA; BioRad, Hercules, CA, USA). Samples were incubated on ice for 30 min followed by short vortexing (10 s, for six times) every 5 min. A total of 40 µL of EV or cell lysates were merged with 8 µL of loading buffer (10% glycerol, 8% sodium dodecyl sulphate, 0.5 M dithiothreitol, and 0.4 M sodium carbonate). This mixture was incubated at 70°C for 5 min. Next, the mixture was loaded onto a NuPAGE+ (Invitrogen, Novex 4%–12% Bis‐Tris) gel and run in an electrophoresis chamber for 120 min at 120 V. Protein transfer was performed onto an iBlot membrane (iBlot 2 Transfer Stacks; Invitrogen) and using the iBlot apparatus (iBlot 2 Dry Blotting System; Invitrogen) for 7 min. This membrane was subjected to incubation with blocking buffer (Odyssey Blocking Buffer; LI‐COR Biosciences, Lincoln, NE, USA) at room temperature for 1 h. Subsequently, the membrane was incubated overnight at 4°C with freshly‐prepared primary antibodies (anti‐CD63: Invitrogen, catalogue number: 10628D,) at a dilution of 1:200, anti‐ALIX (Santa Cruz, catalogue number: sc‐53540,) at a dilution of 1:400, and anti‐ribosomal protein S6 (Cell Signalling Technology, catalogue number: 2217T) at a dilution of 1:2000. Thereafter, the membrane was washed with tris‐buffered saline with Tween (TBS‐T) at 0.1% for 5 min each on a shaker. Next, the membrane was incubated for 60 min with secondary antibodies conjugated with IRDye 800CW (goat anti‐mouse: LI‐COR Biosciences, catalogue number: C00322, and goat anti‐rabbit LI‐COR Biosciences, catalogue number: C90827‐25) that had been diluted to a ratio of 1:10.000. All antibodies were diluted in blocking buffer (Intercept PBS Blocking Buffer, LI‐COR Biosciences, catalogue number: 927–60003). The membrane was washed three times using PBS and revealed using an LI‐COR Odyssey CLx infrared imaging system (LI‐COR Biosciences).

### 2.17 Alkaline phosphatase activity assay

The alkaline phosphatase activity of the chondrocyte‐derived EVs was assessed as previously described (Ansari et al., 2022). A total of 1 × 10^9^ EVs isolated from early‐ or late‐differentiation stages were incubated with 240 µL Triton X‐100 (0.2% v/v in 1X PBS) and MgCl_2_ solution (5 mM in 1X PBS) (Sigma–Aldrich, catalogue number: M1028) for 30 min at room temperature. Next, 80 µL of each sample was combined with 20 µL of 2‐amino‐2‐methyl‐1‐propanol (Sigma–Aldrich, catalogue number: 8.01465; 0.75 M diluted in distilled water) buffer and 100 µL of p‐nitrophenylphosphate (10 mM diluted in distilled water) (Thermo Fisher Scientific, catalogue number: 34045) solution and incubated for 30 min at room temperature. The p‐nitrophenylphosphate was diluted (1X) in diethanolamine substrate buffer (Thermo Fisher Scientific, catalogue number: 34064) before use. The conversion of p‐nitrophenylphosphate to p‐nitrophenol was stopped using NaOH (0.2 M diluted in distilled water) (Sigma–Aldrich, catalogue number: 72068). Absorbance was measured by spectrophotometry (as per 2.8) at 450 nm. Three different batches per time‐point (Day 10 and Day 18) with three technical replicates each were assessed. Since our EV preparation were stored in PBS‐HAT (1X), as described in Section 2.11, we include this buffer as a negative control.

### 2.18 Statistics and reproducibility

**FIGURE 2:**
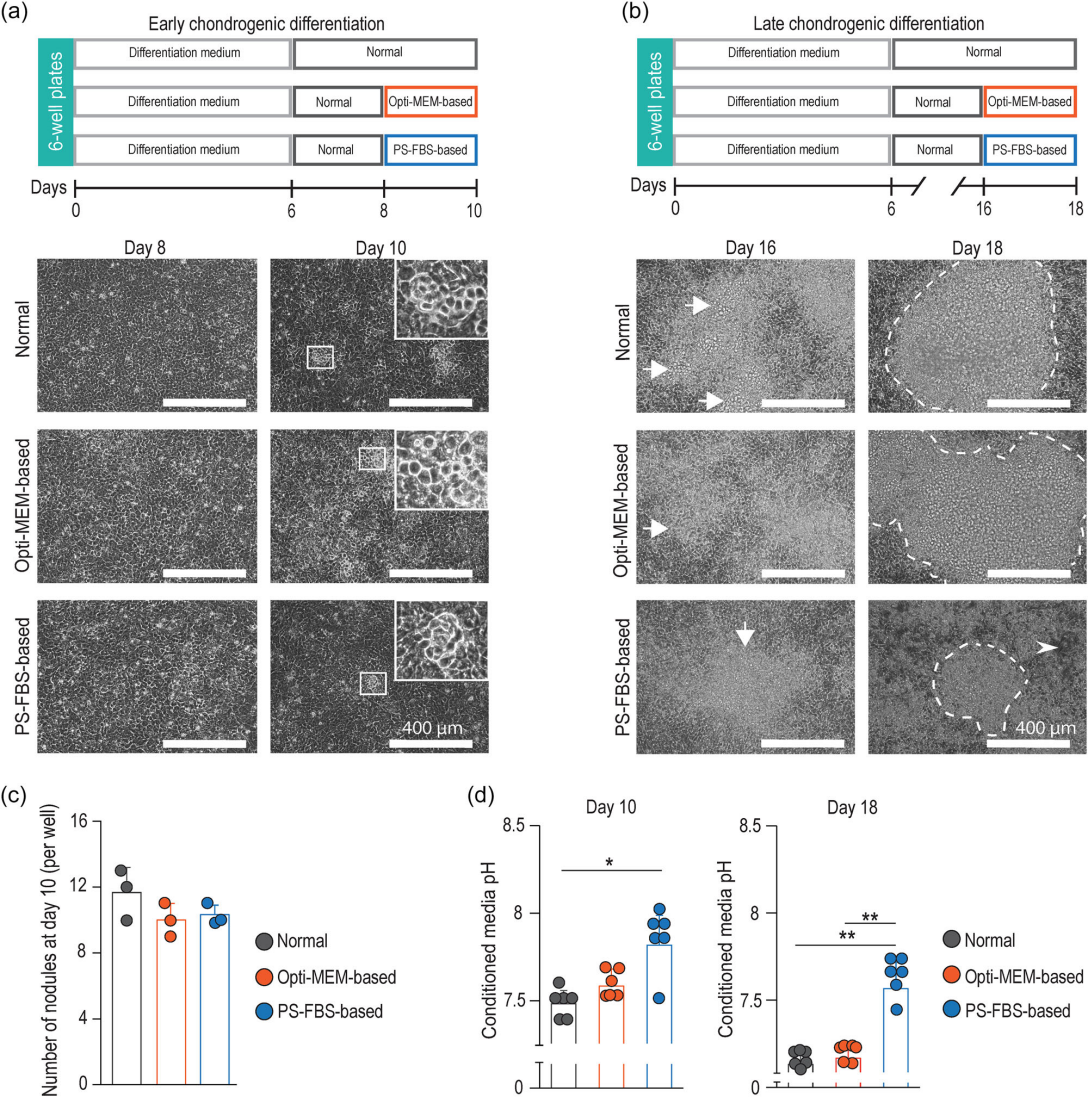
Chondrocyte morphology is maintained when cultured in Opti‐MEM‐based culture medium. (a, b) Time‐line of the experimental setup and morphological characterization of chondrocytes at an early (a) or late (b) differentiation stage. Insets indicate small nodules constituted by round/polygonal cells observed in all conditions at day 10 (a). Cell monolayer showed conjoined and large nodules (white arrows) at day 16 followed by reduction in nodule size (white dotted line) and areas devoid of cells (white arrow head) in the PS‐FBS‐based condition at day 18 (b). (c) Quantification of small nodules observed at day 10. (d) Fluctuations in conditioned media pH during chondrogenic differentiation. Representative microphotographs from two independent differentiation experiments (with nine technical replicates in each experiment). Microphotographs at day 8 and day 16 were documented before changing the culture media. Data in (c) represents three technical replicates from the same differentiation experiment, whereas the datapoints in d derive from two independent differentiation experiments. Data presented as mean ± SD (*p < 0.05. **p < 0.01).

**FIGURE 3:**
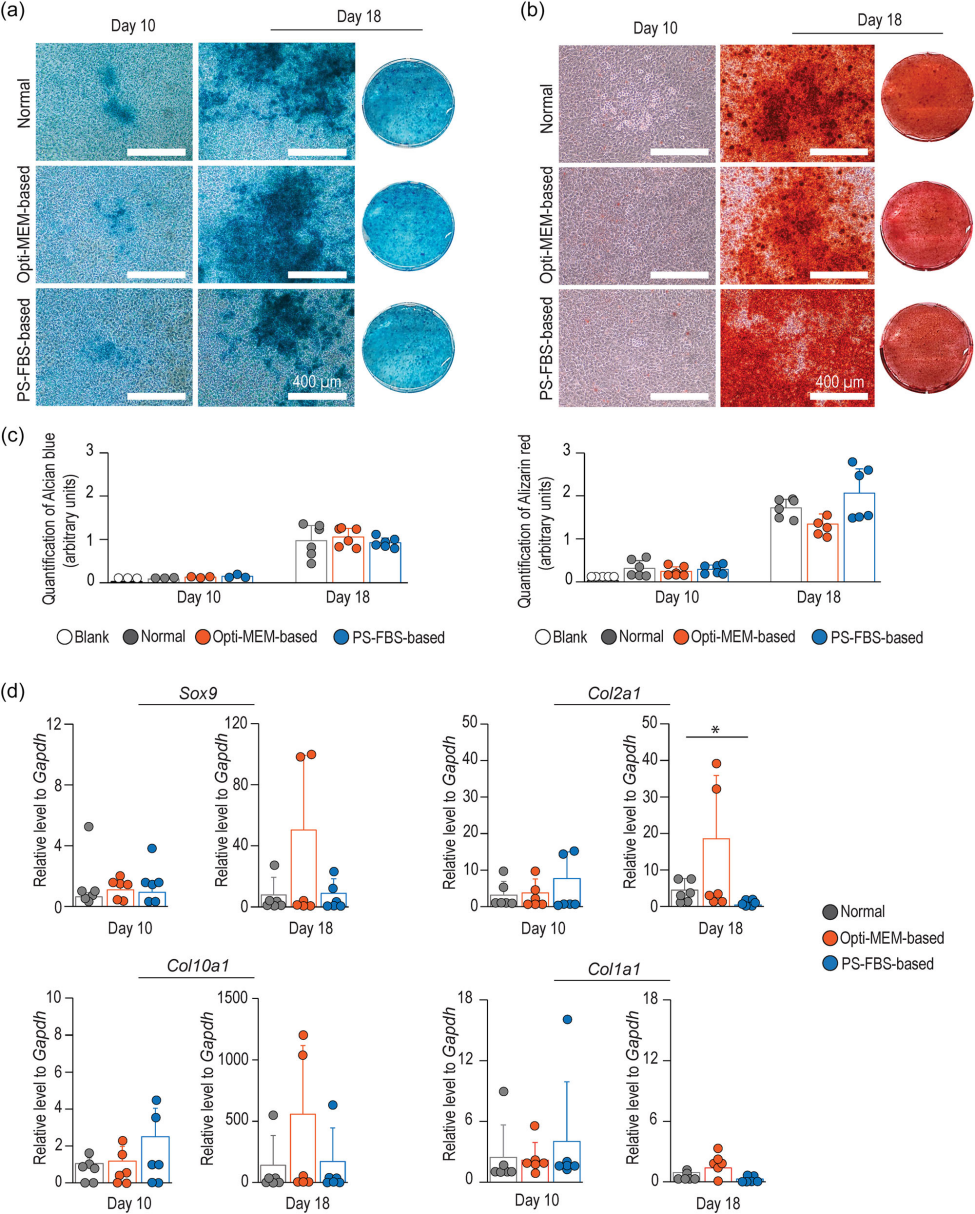
Opti‐MEM‐based culture medium maintains functional capacity and gene expression levels during chondrogenic differentiation. (a–c) Visualization and quantification of proteoglycan levels and matrix mineralization by Alcian blue and alizarin red stainings, respectively. (d) Expression levels of chondrogenic markers assessed by qRT‐PCR. Data in (c) represent six samples from two independent differentiation experiments, except in Alcian blue (day 10), which represent three technical replicates from the same differentiation experiment. All data in (d) derive from three independent experiments. Data presented as mean ± SD (*p < 0.05. **p < 0.01).

All cell culture experiments under different media compositions were performed using at least two independent differentiation experiments each with at least three technical replicates, unless otherwise stated. Regarding the EV isolation, six independent differentiation experiments, referred to as batches, were performed. Each batch was generated from the pooled conditioned medium of five T‐175 flasks, at each time‐point (Day 10 and Day 18). One‐way ANOVA followed by a Tukey's multiple comparison test was applied to determine statistical significance (*p < 0.05, **p < 0.01, ***p < 0.001, and ****p < 0.0001) between experimental groups. All data were verified for normality using the Shapiro–Wilk test. When the ANOVA assumptions (normality) could not be fulfilled (Figure 2d, conditioned media pH; Figure 3c, Alizarin red at D18; Figure 3d, qRT‐PCR experiments), a Kruskal–Wallis test along with a Dunn's test for multiple comparisons was applied (*p < 0.05, **p < 0.01, ***p < 0.001, and ****p < 0.0001). Data are presented in graphs as mean ± standard deviation (SD), with individual data‐points shown. Data normalization is mentioned in figure legends. All statistical analyses were carried out using Rstudio software version 4.1.2 (available on https://cran.r‐project.org/).

### 2.19 Nomenclature

All genes and proteins are referred to according to the HGNC nomenclature system (Seal et al., 2023), except for PDCD6IP, which is referred to by the name familiar to EV researchers: ALIX.

## 3 RESULTS AND DISCUSSION

### 3.1 Opti‐MEM‐based culture medium preserved the chondrogenic differentiation of ATDC5 cells

Upon reaching confluence, ATDC5 cells classically acquire a round phenotype, which is a hallmark of the primordial steps in chondrogenic differentiation (Newton et al., 2012). In line with this observation, our results show that ATDC5 cells transitioned from an elongated to round morphology from day 2 to day 6, respectively (Figure S1a).

Using the normal culture medium as a guide, we devised two different chondrogenic EV‐depleted media, based on (i) serum‐free Opti‐MEM (Karttunen et al., 2022) and (ii) pre‐spun FBS (PS‐FBS) (Eitan et al., 2015) (Table 1). Cells were grown in Normal medium, which was changed to the EV‐depleted media in the final 48 h of culture (Figure 2a,b). To compare the culture media based on their impact on cell morphology, we examined ATDC5 cultures under light microscopy at early‐ and late‐differentiation stages. At an early differentiation stage (Figure 2a), the cell monolayer did not show major morphological variations. All conditions harboured small nodules formed by round/polygonal cells in similar numbers (Figure 2a,c). As expected, at a late differentiation stage (Figure 2b), we observed conjoined and large nodules with a translucent ECM in Opti‐MEM‐based cultures, which were comparable to the Normal cultures (Figures 2b and S1b). By contrast, we noticed a reduction in nodule size with irregular areas devoid of cells in the PS‐FBS‐based condition, indicating that PS‐FBS‐based medium led to morphological abnormalities. Together, these results demonstrate that chondrocytes maintained a typical phenotype when grown in Opti‐MEM‐based culture medium. Similarly, morphology is conserved in mesenchymal stromal cells (MSCs) (Karttunen et al., 2022), human pluripotent stem cells (hPSCs) (Karttunen et al., 2022) and a variety of cell‐lines, including N2a (Li et al., 2015), HeLa (Bost et al., 2022) and HEK (Bost et al., 2022) cells, when cultured with Opti‐MEM (Karttunen et al., 2022).

Recent reports have revealed that pH strongly influences EV biology in cell cultures; for example, a neutral or acidic pH may alter EV concentration, integrity, cargo sorting, release and uptake (Cheng et al., 2019; Nakase et al., 2021). We firstly confirmed that the pH levels of the chondrogenic EV‐depleted media were similar to that of Normal medium when freshly‐prepared (Figure S2a). However, when we measured the pH after culture, the resultant conditioned media from both Normal and Opti‐MEM‐based cultures had similar pH levels at day 10 and day 18, whereas the PS‐FBS cultures revealed higher pH levels at both time‐points (Figure 2d). This observation suggests that PS‐FBS‐based medium may be ill‐suited for collecting relevant populations of chondrocyte‐derived EVs.

To further study the functional maturation of chondrocytes, we performed histological staining with Alcian blue and alizarin red (Figure 3a–c). As expected, low levels of ECM deposition and an absence of mineralization were observed at an early differentiation stage (Newton et al., 2012). In contrast, extensive Alcian blue‐positive areas were present in all conditions at a late differentiation stage (day 18), confirming that cells underwent chondrogenesis and assembled a glycosaminoglycan (GAG)‐rich ECM. Moreover, at this time‐point, ECM mineralization was observed, as reflected by widespread areas positive for alizarin red. However, PS‐FBS‐based medium showed a mild increase (*p *> 0.05) in mineralization compared to Normal and Opti‐MEM‐based cultures, respectively (Figure 3c).

Growth plate chondrocytes express well‐established markers, including *Sox9*, *Col2a1* and, at later differentiation stages, *Col10a1* (Kozhemyakina et al., 2015). Using these markers, we applied qRT‐PCR to assess the cell differentiation stage after culture. In comparison with day 10, all markers were elevated by day 18, but no statistical differences were observed between culture conditions within each time‐point, except for *Col2a1*, which was drastically reduced in the PS‐FBS‐based conditions (Figure 3d). Moreover, very low levels of *Col1a1* transcripts were observed at both time points (Figure 3d), supporting the notion that chondrogenic differentiation followed the expected course, and that withdrawal of Normal medium did not promote de‐differentiation of the cells (Chen et al., 2022).

### 3.2 Opti‐MEM‐based culture medium maintained cell viability during chondrogenic differentiation

**FIGURE 4:**
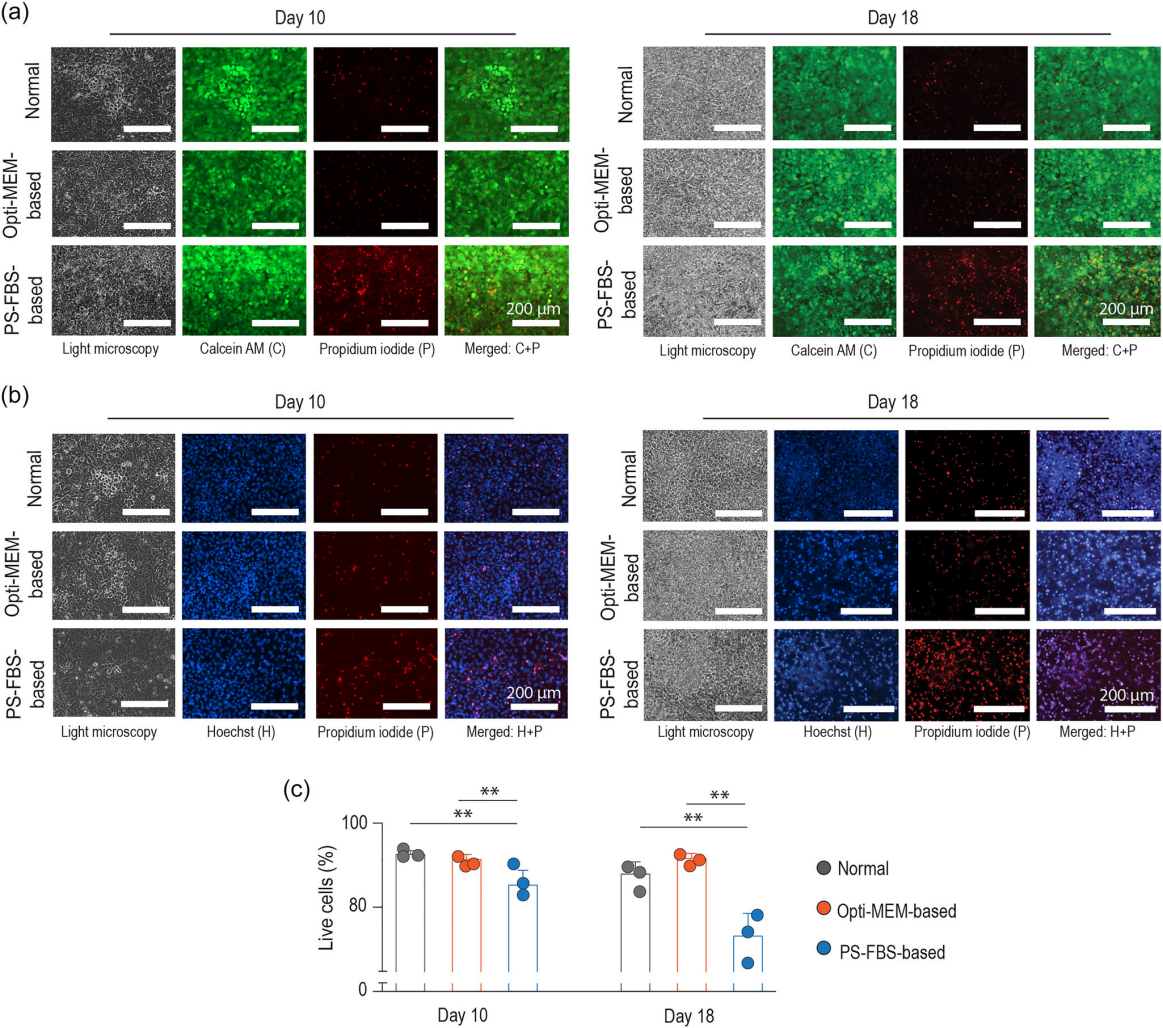
Chondrocyte viability is preserved by Opti‐MEM‐based culture medium at early‐ and late‐differentiation stages. Fluorescence microscopy of Calcein AM+propidium iodide (a) and Hoechst+propidium iodide (b) at day 10 and day 18. (c) Quantitative analysis of live cells (%) by Hoechst+propidium iodide assessment (mean ± SD, *n* = 3 from one differentiation. **p < 0.01).

Due to their overlapping sizes, apoptotic bodies cannot be easily separated from exosomes and microvesicles in EV preparations, and are generally regarded as contaminants (Mathieu et al., 2019) Therefore, we interrogated the extent of cell death in our culture systems as outlined in the minimal experimental requirements defined by the International Society for Extracellular Vesicles (MISEV 2018) (Théry et al., 2018). Initially, we performed an exploratory analysis using calcein AM and propidium iodide (Figure 4a). The detection of intracellular Calcein AM fluorescence revealed that the cell monolayer was substantially formed of live cells in the Normal and Opti‐MEM‐based culture medium at early‐ or late‐differentiation stages (Figures 4a and S2b). By contrast, cells grown under the PS‐FBS‐based culture medium exhibited a marked increase in cell death (Figures 4a and S2b).

We then confirmed that Opti‐MEM‐based culture medium maintained cell viability during chondrogenic differentiation by quantifying the proportion of dead cells (propidium iodide‐positive) as a percentage of all cells (Hoechst positive; Figure 4b,c). When small nodules (Figure 2a) were monitored for viability, those in Normal and Opti‐MEM‐based culture media contained more live cells than PS‐FBS‐based culture medium (Figure S2c).

Altogether, our data show that chondrocytes cultured in Opti‐MEM‐based culture medium recapitulated fundamental steps in endochondral ossification including proteoglycan synthesis, matrix mineralization and expression of chondrogenic markers along with maintaining viable cells. Thus, we selected the Opti‐MEM‐based culture medium to scale‐up for chondrocyte‐derived EV yielding (Figure 1).

### 3.3 Scale‐up of ATDC5 culture did not impair chondrogenic differentiation or viability

**FIGURE 5:**
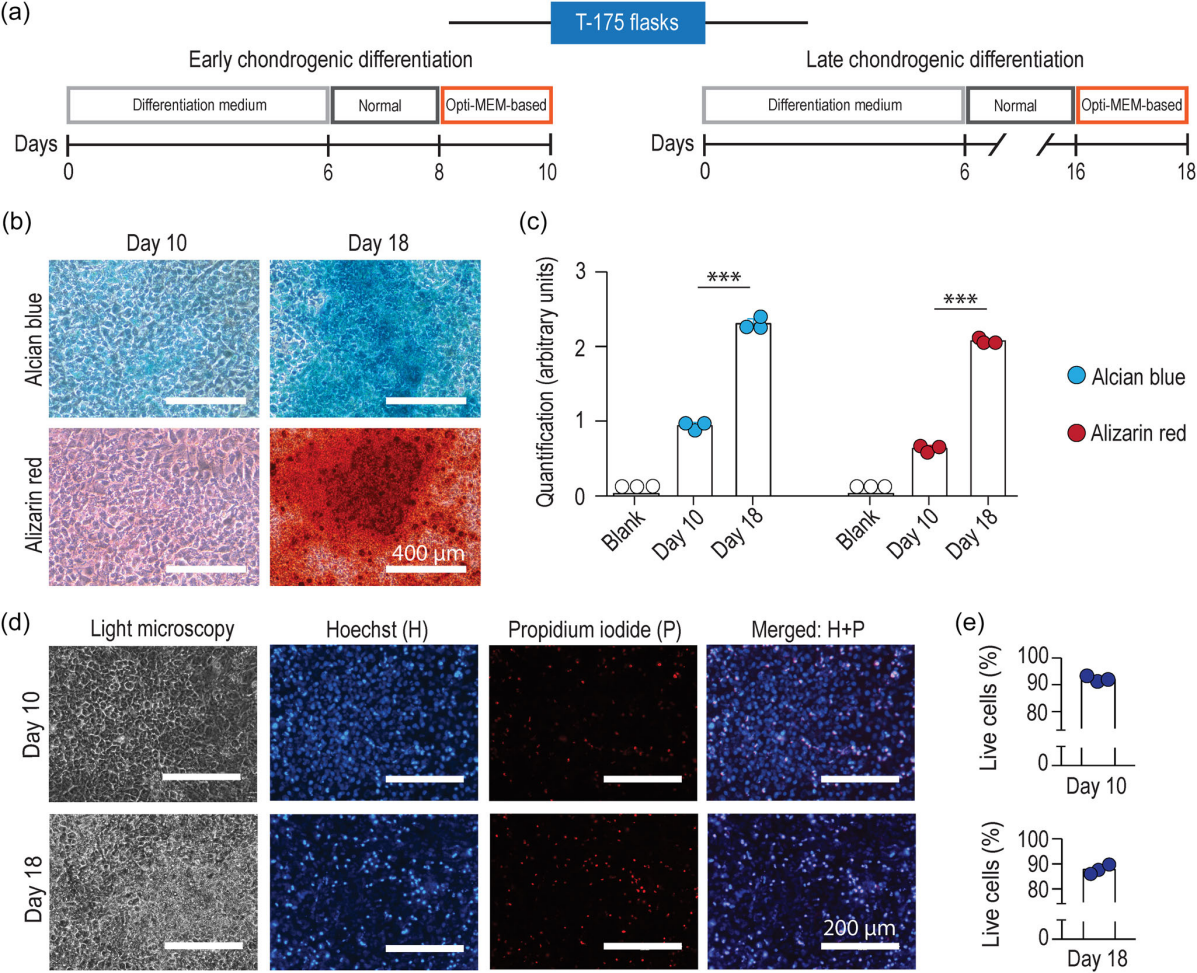
Chondrogenic differentiation is preserved by Opti‐MEM‐based culture medium in large‐scale EV production. (a) Using T‐175 culture flasks, ATDC5 cells were differentiated in normal medium, which was changed to the Opti‐MEM‐based medium in the final 48 h of culture. (b) Histological assessment of proteoglycan production (Alcian blue) and mineralization (Alizarin red). (c) Quantification of Alcian blue and Alizarin red staining. (d–e) Chondrocyte viability assessed with Hoechst+propidium iodide. Each datapoint was produced from an independent differentiation experiment (mean ± SD. ***p < 0.001).

Large‐scale EV production is a crucial aspect in EV research, particularly in preclinical models. For instance, large numbers of particles are required to interrogate the EV bioactivity *in vivo* (∼1×10^9^ – 1×10^12^ per mouse) (Gupta et al., 2021). Further, increased production of EVs also demands careful consideration of the cell culture conditions (Wiklander et al., 2019). Accordingly, with the aim of increasing the volume of conditioned medium we could collect from each culture, and thereby EV yield, we grew ATDC5 cells in Opti‐MEM‐based culture medium using T‐175 flasks (Figures 1 and 5a). We firstly tested whether culturing the cells in larger flasks affected the chondrogenic differentiation process: the cell monolayer showed round/polygonal cells and nodules that increased in size from day 10 to day 18 (Figure S3a). The pH of the conditioned medium decreased at a late differentiation stage (Figure S3b), as initially observed in culture plates (Figure 2d). Moreover, Alcian blue and alizarin red staining confirmed that cells underwent expected chondrogenic differentiation at both time‐points (Figure 5b,c). When assessing cell viability, chondrocyte cultures were characterized by high percentages of live cells at both time‐points (Figure 5d,e). These findings corroborate the observations that chondrogenic Opti‐MEM‐based medium sustained chondrocyte properties and, therefore, is suitable for EV production.

**FIGURE 6:**
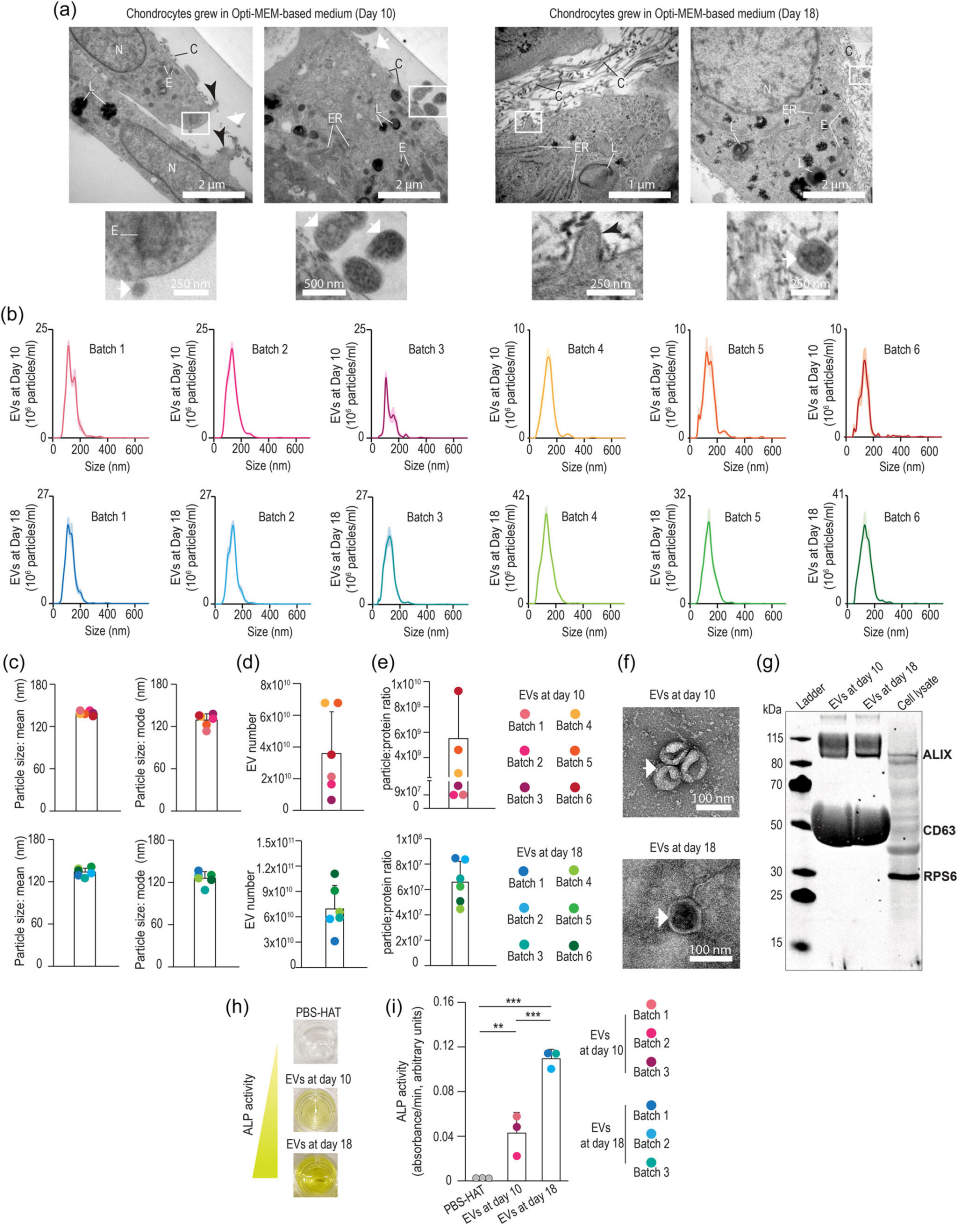
Chondrocytes grown in Opti‐MEM‐based culture medium and T‐175 flasks produce abundant EVs. (a) ultrastructural characterization of chondrocytes at day 10 and day 18. Small (<200 nm) and large (>200 nm) EVs (white arrows) were observed in close proximity to the plasma membrane or embedded in the collagen matrix. Chondrocytes showed outward budding of the plasma membrane (black arrowheads) and vesicle‐like structures close to the plasma membrane. (b) NTA of each batch indicates reproducible particle size distributions. (c) NTA was used to calculate the sizes of chondrocyte‐derived EVs. (d) The ratio of particles:protein was calculated and is presented as number of particles per µg protein. (e) Number of particles quantified by NTA. (f) Representative chondrocyte‐derived EVs were imaged by TEM. (g) Chondrocyte‐derived EV preparations were analysed by western blot. Preparations contained EV markers (CD63 and ALIX) whereas a potential contaminant from the cytoplasm (RPS6) was only detected in the cell lysate. (h–i) Chondrocyte‐derived EVs from late‐differentiation stages have elevated alkaline phosphatase activity. C, collagen fibres are indicated for navigation; E, endosome‐like vesicle; ER, rough endoplasmic reticulum; L, lysosome; N, nucleus.

To visualize the chondrocyte morphology at an ultrastructural level, we used transmission electron microscopy (TEM) (Figure 6a). At both differentiation stages assessed, nuclei were typically defined by a nuclear envelope and harboured mild electron‐dense nucleoli‐like structures with absence of chromatin condensation. The cytosol revealed heavily electron‐dense cell processes and organelles, including rough endoplasmic reticulum, lysosomes and endosomal‐like vesicles. Importantly, several endosome‐like structures were observed in close proximity to the inner leaflet of the plasma membrane. Notably, chondrocytes showed outward budding of the plasma membrane with small (<200 nm) and large (>200 nm) EVs (Théry et al., 2018) in the extracellular space at both time‐points. These results are in line with pioneering ultrastructural observations of the growth plate wherein chondrocytes were shown to release EVs (Anderson, 1969; Bonucci, 1967). Likewise, clusters of EVs close to the cell surface have been observed in other cell‐lines such as HeLa and B cells (Edgar et al., 2016). Hence, our data indicate that chondrocytes grown in Opti‐MEM‐based medium had the functional machinery to generate and release EVs.

### 3.4 Nanoparticles obtained from scaled‐up production were confirmed as chondrocyte‐derived EVs

We applied SEC, with an optimum particle isolation range of 70–1000 nm, in order to isolate EVs from the conditioned medium obtained from six independent differentiation experiments (batches) of five T‐175 flasks at both early‐ and late‐differentiation stages (Figure 5a). The sizes of particles obtained from SEC were quantified by NTA (Figure 6b,c), revealing particle sizes predominantly in the range of small EVs (<200 nm) in all conditions. We used the number of particles obtained from NTA to calculate the total number of EVs obtained per ATDC5 culture batch (Figure 6d). These quantities make it feasible that each batch of EVs could be used as an experimental replicate in functional studies (Gupta et al., 2021; Hagey et al., 2023; Veerman et al., 2021). Since every batch is comprised of five T‐175 flasks, we used those values to calculate the number of EVs obtained from every square cm of the culture flask: 2.5 × 10^7^ particles/cm^2^ (batch 1), 2 × 10^7^ particles/cm^2^ (batch 2), 7.8 × 10^6^ particles/cm^2^ (batch 3), 3.9 × 10^7^ particles/cm^2^ (batch 4), 3.9 × 10^7^ particles/cm^2^ (batch 5), 2.0 × 10^7^ particles/cm^2^ (batch 6) at Day 10, and consistently higher yields per batch at Day 18 of 3 × 10^7^ particles/cm^2^ (batch 1), 6 × 10^7^ particles/cm^2^ (batch 2), 6 × 10^7^ particles/cm^2^ (batch 3), 7.5 × 10^7^ particles/cm^2^ (batch 4), 1.1 × 10^8^ particles/cm^2^ (batch 5), and 1.3 × 10^8^ particles/cm^2^ (batch 6). By contrast, the number of particles detected in the EV‐storage buffer, PBS‐HAT, was lower (2.6 particles per frame) than the detection threshold (30–120 particles per frame) of the NTA, and there was an absence of EV‐like particles in PBS‐HAT when analysed by TEM (Figure S4).

In recent years, EV researchers have used particle:protein ratio as a way to assess the purity of EV preparations (Buzas, 2022). Previously, non‐EV‐associated proteins were considered contaminants of EV preparations (Webber & Clayton, 2013); however, more recent research has revealed that at least some co‐isolated proteins can coat EVs, to form a so‐called protein corona, and are integral for EV function (Tóth et al., 2021; Wolf et al., 2022). Here, we observed that chondrocyte‐derived EVs isolated at an early differentiation stage had similar particle‐to‐protein ratios among batches (batch 1 = 9.5 × 10^7^ particles/µg protein, batch 2 = 9 × 10^7^ particles/µg protein, batch 3 = 1 × 10^8^ particles/µg protein, batch 4 = 2.7 × 10^9^ particles/µg protein, batch 5 = 4.6 × 10^9^ particles/µg protein, batch 6 = 9.3 × 10^9^ particles/µg protein, Figure 6e). Furthermore, chondrocyte‐derived EVs isolated at a late‐differentiation stage were also consistently similar in terms of particle‐to‐protein ratio (batch 1 = 8.5 × 10^7^ particles/µg protein, batch 2 = 8.4 × 10^7^ particles/µg protein, batch 3 = 6.9 × 10^7^ particles/µg protein, batch 4 = 5 × 10^7^ particles/µg protein, batch 5 = 4.6 × 10^7^ particles/µg protein, batch 6 = 6.3 × 10^7^ particles/µg protein). The high levels of reproducibility at each time‐point indicate the robustness of the protocol (Figure 6e).

To verify the identity of these nanoparticles, we applied negative stain TEM, in which EVs are characteristically identified as cup‐shaped particles (Veerman et al., 2021). In line with this general observation, the ultrastructural image of chondrocyte‐derived EVs showed particles delimited by a plasma membrane and a classical cup‐shaped morphology (Figure 6f).

As an additional validation method, we assessed the presence of conventional EV markers using western blot (Figure 6g). In accordance with the MISEV 2018 guidelines (Théry et al., 2018), we selected three main categories of EV marker: CD63 (as a transmembrane or glycosylphosphatidylinositol [GPI]‐anchored protein, category 1) (Théry et al., 2018), ALIX (as a cytosolic protein recovered in EVs, category 2, also known as PDCD6IP) (Théry et al., 2018) and ribosomal protein S6 (RPS6; as a major constituent of non‐EV co‐isolated structures, category 3) (Théry et al., 2018). Chondrocyte‐derived EV preparations were highly enriched with transmembrane (CD63) and intraluminal (ALIX) EV‐markers, but did not contain contamination in the form of RPS6.

### 3.5 Chondrocyte‐derived EVs harbour mineralizing properties

Based on our analysis of chondrocyte differentiation and ECM mineralization, we considered that the EVs deriving from our early‐differentiation stage (i.e., from medium conditioned during days 8–10 of culture) reflected EVs released by chondrocytes before the onset of ECM mineralization, whereas the late‐differentiation stage (i.e., medium conditioned during days 16–18 of culture) reflected EVs released by chondrocytes during cartilage mineralization. To test functional differences between the chondrocyte‐derived EV populations collected at both time‐points, we quantified their alkaline phosphatase (ALP) activities. ALP is an ecto‐enzyme that is found on the outer surface of cartilage matrix vesicles, which facilitates ECM mineralization by degrading pyrophosphate (a potent inhibitor of mineralization) and cleaving inorganic phosphate from near‐lying molecules for use in hydroxyapatite nucleation and propagation (Cui et al., 2016). ALP is enriched in EVs within the hypertrophic zone of the growth plate *in vivo* (Matsuzawa & Anderson, 1971). Of note, chondrocyte‐derived EVs isolated from the late‐differentiation stage had higher levels of ALP activity compared to those EVs derived from the early‐differentiation stage (Figure 6i,j). Therefore, we obtained two different populations of chondrocyte‐derived EVs that reflect the populations of EVs found in the growth plate *in vivo*, both before and during mineralization.

Altogether, our results demonstrate that chondrogenic Opti‐MEM‐based culture medium combined with SEC can be used to recover EVs from chondrocytes at both early‐ and late‐differentiation stages of relatively small size, with typical ultrastructure markers, and mineralizing activity, in quantities suitable for downstream experimentation.

## 4 CONCLUSION

In summary, we found that the chondrogenic ATDC5 cell‐line produced a substantial amount of EVs when grown in a chondrogenic Opti‐MEM‐based culture medium at two differentiation stages. EV isolation was achieved without impairing chondrocyte differentiation or viability, thereby enabling the recovery of high‐quality chondrocyte‐derived EV populations. By isolating EVs from cells at early and late stages of endochondral ossification, the model provides a way to obtain EVs representative of those released by chondrocytes before and during ECM mineralization, respectively. Hence, our findings provide researchers with a tool to generate chondrocyte‐derived EVs, which we hope will facilitate future research to improve our fundamental understanding of the roles of EVs during endochondral bone growth and fracture repair.

## AUTHOR CONTRIBUTIONS

Jose G. Marchan‐Alvarez: Conceptualization (supporting); data curation (lead); formal analysis (lead); investigation (lead); methodology (lead); project administration (lead); validation (lead); visualization (lead); writing—original draft (lead); writing—review and editing (equal). Loes Teeuwen: Data curation (supporting); formal analysis (supporting); methodology (supporting); software (supporting). Doste R. Mamand: Data curation (supporting); formal analysis (supporting). Susanne Gabrielsson: Conceptualization (supporting); formal analysis (supporting); investigation (supporting); methodology (supporting); resources (supporting); writing—review and editing (supporting). Klas Blomgren: Investigation (supporting); project administration (supporting); supervision (supporting); writing—review and editing (supporting). Oscar P.B. Wiklander: Conceptualization (supporting); formal analysis (supporting); investigation (supporting); methodology (supporting); resources (supporting); supervision (supporting); writing—review and editing (supporting). Phillip T. Newton: Conceptualization (lead); formal analysis (supporting); funding acquisition (lead); project administration (supporting); resources (supporting); supervision (lead); visualization (supporting); writing—original draft (supporting); writing—review and editing (supporting).

## CONFLICT OF INTEREST STATEMENT

Susanne Gabrielsson has a patent on B cell‐derived EVs in immune therapy and holds a position on the scientific advisory board of Anjarium Biosciences. Oscar P.B. Wiklander has a financial interest in Evox Therapeutics.

## Supporting information

Supplementary Information

Supplementary Information

Supplementary Information

Supplementary Information

## Data Availability

The data that support the findings of this study are available upon request.
