# Supplementary figures and images for: A protocol to differentiate the chondrogenic ATDC5 cell‐line for the collection of chondrocyte‐derived extracellular vesicles
Source: J Extracell Biol. 2024 Sep 5;3(9):e70004. doi: 10.1002/jex2.70004 (PMC11375531; doi:10.1002/jex2.70004)

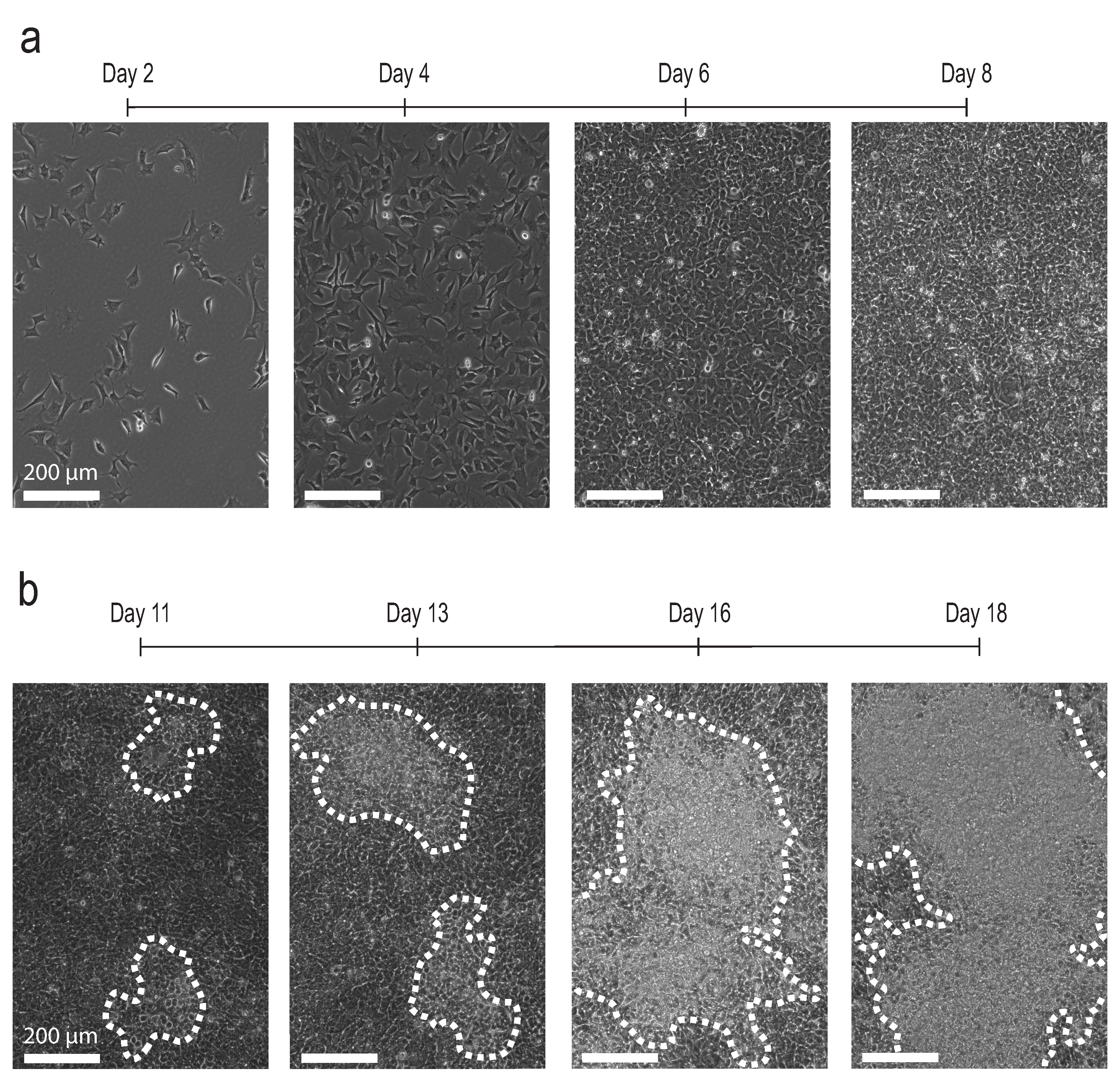

Supplement: Supplementary file 1 — Supplementary Information [file JEX2-3-e70004-s003.tif]

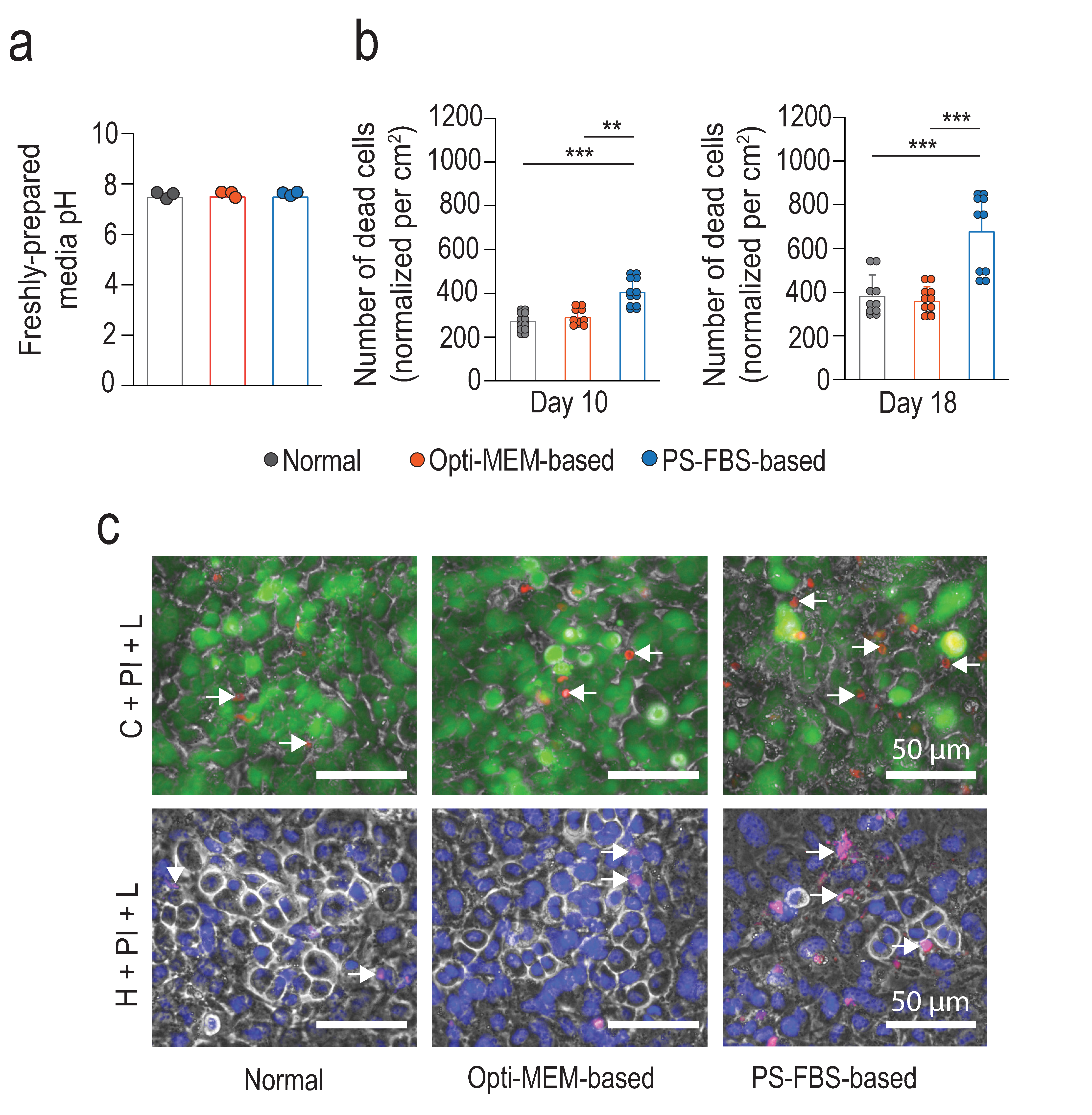

Supplement: Supplementary file 2 — Supplementary Information [file JEX2-3-e70004-s002.tif]

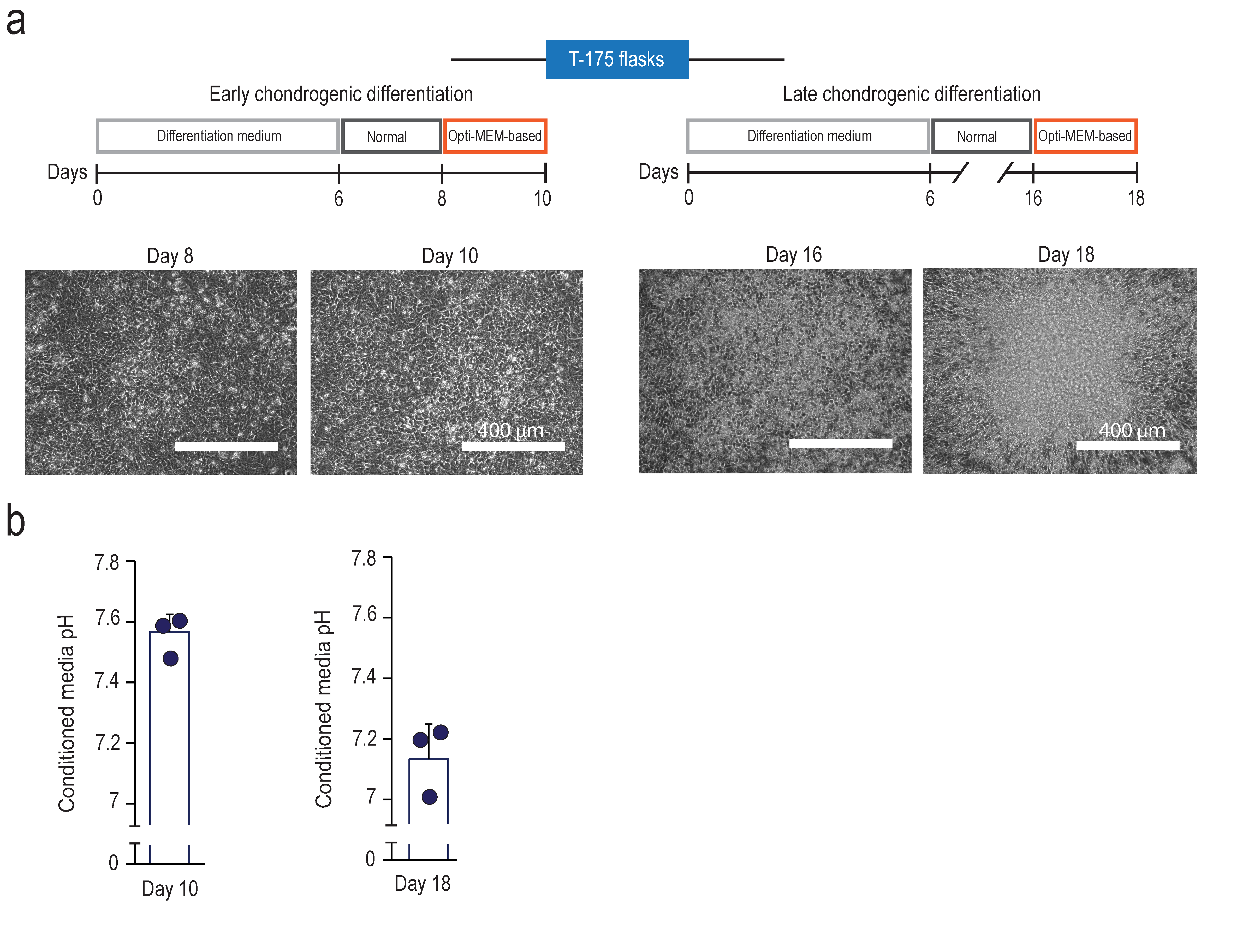

Supplement: Supplementary file 3 — Supplementary Information [file JEX2-3-e70004-s004.tif]

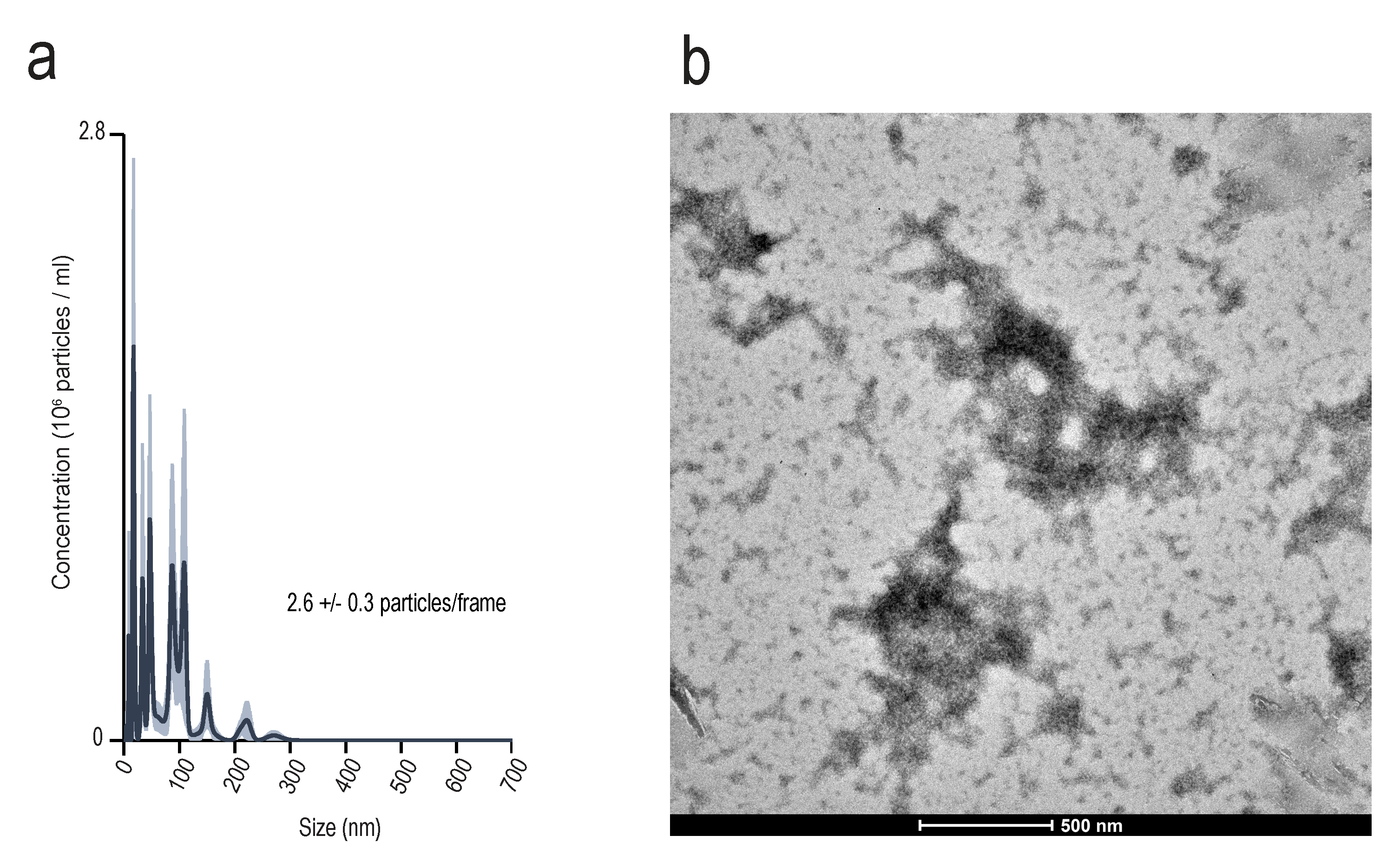

Supplement: Supplementary file 4 — Supplementary Information [file JEX2-3-e70004-s001.tif]
